# Methodological Characteristics of Clinical Trials Supporting the Marketing Authorisation of Advanced Therapies in the European Union

**DOI:** 10.3389/fphar.2021.773712

**Published:** 2021-11-29

**Authors:** Carolina Iglesias-Lopez, Antònia Agustí, Antonio Vallano, Merce Obach

**Affiliations:** ^1^ Department of Pharmacology, Therapeutics and Toxicology, Universitat Autònoma de Barcelona, Barcelona, Spain; ^2^ Clinical Pharmacology Service, Vall d’Hebron University Hospital, Barcelona, Spain; ^3^ Medicines Department, Catalan Healthcare Service, Barcelona, Spain

**Keywords:** drug development, drug approval, research design, methods, clinical trials, advanced therapies, cell- and tissue-based therapy, genetic therapy

## Abstract

Several advanced therapy medicinal products (ATMPs) have been approved in the European Union (EU). The aim of this study is to analyse the methodological features of the clinical trials (CT) that supported the marketing authorization (MA) of the approved ATMPs in the EU. A systematic review of the characteristics of pivotal CT of ATMPs approved in the EU until January 31st, 2021 was carried out. A total of 17 ATMPs were approved and 23 CT were conducted to support the MA (median, 1, range, 1–3). Of those studies, 8 (34.78%) were non-controlled and 7 (30.43%) used historical controls. Only 7 (30.4%) were placebo or active-controlled studies. Among all CT, 21 (91.3%) were open-label and 13 (56.52%) had a single-arm design. To evaluate the primary endpoint, 18 (78.26%) studies used an intermediate and single variable. The median (IQR) number of patients enrolled in the studies was 75 (22–118). To date, ATMPs’ approval in the EU is mainly supported by uncontrolled, single-arm pivotal CT. Although there is a trend toward an adaptive or a life cycle approach, a switch to more robust clinical trial designs is expected to better define the benefit and the therapeutic added value of ATMPs.

## Introduction

Advanced therapy medicinal products (ATMPs) are a medicinal class that includes gene, cell and tissue therapies. The success of ATMP development and the approval of these therapies in the European Union (EU) has been crucial to the growth of clinical research during the last few years in this field, particularly for gene therapy.

Multiple indications are being targeted, most of them being refractory and recurrent stages of a disease that lacks effective therapeutic alternatives, and a significant proportion of them affecting the paediatric population (Alamo et al., 2019). With the introduction of ATMPs that can cover unmet needs and have the potential to cure life-threatening diseases, biological therapies initiated a shift from traditional clinical development pathway to an accelerated and highly product-specific one. The adaptive pathway concept and Priority Medicines scheme (PRIME) were launched in the EU specifically to speed the access of products targeting a significant unmet medical need. Several approved ATMPs were granted a PRIME designation and accelerated marketing authorisation application assessment during their development, allowing early access to these medicines (Iglesias-Lopez et al., 2021a).

Due to the type of target diseases, the inherent complexity of these products, and their accelerated developments, less comprehensive clinical data might be generated. These characteristics may lead to uncertainties in the benefit/risk profile for the product at the time of marketing authorization (MA). The aim of this study is to further analyse the clinical development of the current approved ATMPs’. Here, we describe the methodological features of the clinical trials that have driven ATMPs to their European approval and we compare the gene therapy trials versus the cell and tissue engineered trials.

## Methods

A systematic review of the pivotal trials’ features that supported the MA of the ATMPs approved in the EU was carried out using the following approach:

1) *Search strategy*: Data collection was primarily extracted from European Public Assessment Reports on the European Medicines Agency (EMA) website (www.ema.europa.eu). The search was carried out until January 31st, 2021. In addition, a search for the main clinical trials of the approved ATMPs was conducted using ClinicalTrials.gov database and the related publications.
2) *Eligibility criteria*: Only products classified as ATMPs according to the EMA criteria (European Medicines Agency, 2010; Iglesias-Lopez et al., 2019) and authorised under centralised procedure in the EU have been considered. Combined ATMPs class, i.e., ATMP combined with a medical device, have been grouped according to the main ATMP category: gene therapy medicinal product, somatic cell therapy medicinal products or tissue engineered products. Only those trials identified or referenced as pivotal, and therefore, decisive for the MAA were analysed.
3) *Data extraction and collected variables*: The authors designed specific data extraction forms using Excel 2019 (Microsoft Corporation, Redmond, WA, United States) to collect information. For each ATMP the following variables were collected: type of ATMP, pharmacotherapeutic group, ATC code, therapeutic area (according to MeSH terms), diseases and other circumstances for its use (according to chapter’s title from the international version of the ICD-10), number of assessed clinical indications and pivotal clinical trials conducted. For each pivotal clinical trial, the following variables were selected: phase, design, type of randomization, type of control, type of study blinding, number of arms, participating centres, type of hypothesis and primary endpoint, presence and type of health-related quality of life (HRQoL) endpoints, presence of pre-specified analysis, duration of the main phase of the study, pivotal trial ongoing at the time of MAA, overall number of patients that participated in the study (enrolled, on intervention arm or control arm and safety set), age and sex of population, existence or absence of previous treatments, and geographic location of the pivotal trial. To determine if the study was ongoing at the time of the submission, the MAA submission date and the final data collection date for the primary outcome measure of the pivotal clinical trial were reviewed. Standard definitions of analysis set were used to classify among intended to treat (ITT), modified ITT (mITT) and per protocol set (PP) following ICH (E9) and EMA guidelines (ICH, 1998b; European Medicnes Agency, 2007). To assign the type of hypothesis in the case of two variables being used to evaluate the primary endpoint, the most robust variable was selected, i.e., final *versus* surrogate variables.
4) *Statistical analysis*: Statistical analysis for categorical and continuous variables was made using means of proportions, mean, standard deviation (SD), median, quartiles 25 and 75 (Q25, Q75), and range (minimum and maximum). The statistical analysis was performed using SAS® 9.4 (SAS Institute Inc., Cary, NC, United States).

## Results

A total of 17 ATMPs have been approved in the EU (Table 1) and 23 main trials were conducted to support the MA for these products (median, 1, range, 1–3). The ATMPs trials by disease area, according to ICD-10 classification, included: neoplasms (7), endocrine, nutritional and metabolic diseases (2), diseases of the blood and blood-forming organs and certain disorders involving the immune mechanism (2), diseases of the eye and adnexa (2), diseases of the nervous system (1), diseases of the musculoskeletal system and connective tissue (3), diseases of the digestive system (1). In addition, there were 6 ATMPs for rare inherited disorders and 6 for neoplasms in which 4 were indicated for haematological malignancies and 2 for solid tumours. The detailed results of this study are presented in Table 2 by type of ATMP, in Table 3 for gene therapy studies and in Table 4 for cell and tissue therapy studies.

**TABLE 1:** Approved ATMPs in the European Union and therapeutic indication.

Trade name	International non-proprietary name (INN) or common name	Pharmacotherapeutic group/ATC code	Therapeutic area (MeSH)	Chapter’s title from the international version of the ICD-10
Gene therapy medicinal products				
Kymriah®	Tisagenlecleucel	Antineoplastic agents/L01XX71	Precursor B-Cell Lymphoblastic Leukemia-Lymphoma	Neoplasms
Kymriah®	Tisagenlecleucel	Antineoplastic agents/L01XX71	Lymphoma, Large-B-cell, Diffuse	Neoplasms
Yescarta®	Axicabtagene ciloleucel	Antineoplastic agents/L01XX70	Lymphoma, Large-B-cell, Diffuse	Neoplasms
Tecartus®	Autologous peripheral blood T cells CD4 and CD8 selected and CD3 and CD28 activated transduced with retroviral vector expressing anti-CD19 CD28/CD3-zeta chimeric antigen receptor and cultured	Antineoplastic agents/L01X	Lymphoma, Mantle-Cell	Neoplasms
Imlygic®	Talimogene laherparepvec	Antineoplastic agents/L01XX51	Melanoma	Neoplasms
Glybera®	Alipogene tiparvovec	Lipid modifying agents/C10AX10	Hyperlipo-proteinemia type I	Endocrine, nutritional and metabolic diseases
Strimvelis®	Autologous CD34 ^+^ enriched cell fraction that contains CD34 ^+^ cells transduced with retroviral vector that encodes for the human ADA cDNA sequence	Immunostimulants/L03	Severe combined immunodeficiency	Diseases of the blood and blood-forming organs and certain disorders involving the immune mechanism
Luxturna®	Voretigene neparvovec	Ophthalmologicals, other ophthalmologicals/S01XA27	Leber congenital amaurosis Retinitis Pigmentosa	Diseases of the eye and adnexa
Zynteglo®	Betibeglogene autotemcel	Other haematological agents/B06AX02	Beta-Thalassemia	Diseases of the blood and blood-forming organs and certain disorders involving the immune mechanism
Zolgensma®	Onasemnogene abeparvovec	Other drugs for disorders of the musculoskeletal system/M09AX09	Muscular Atrophy Spinal	Diseases of the nervous system
Libmeldy®	Atidarsagene autotemcel	Other nervous system drugs/N07	Leukodystrophy, Metachromatic	Endocrine, nutritional and metabolic diseases
Somatic-cell therapy medicinal products				
Provenge®	Autologous peripheral-blood mononuclear cells activated with prostatic acid phosphatase granulocyte-macrophage colony-stimulating factor (Sipuleucel-T)	Other immunostimulants/L03AX17	Prostatic Neoplasms	Neoplasms
Zalmoxis®	Allogeneic T cells genetically modified with a retroviral vector encoding for a truncated form of the human low affinity nerve growth factor receptor (ΔLNGFR) and the herpes simplex I virus thymidine kinase (HSV-TK Mut2)	Antineoplastic agents/L01	Hematopoietic Stem Cell Transplantation	Neoplasms Factors influencing health status and contact with health services
			Graft vs Host disease	
Alofisel®	Darvadstrocel	Immunosuppressants/L04	Rectal Fistula	Diseases of the digestive system
Tissue-engineered medicinal products				
Chondrocelect®	Characterised viable autologous cartilage cells expanded *ex vivo* expressing specific marker proteins/	Other drugs for disorders of the musculoskeletal system/M09AX02	Cartilage Diseases	Diseases of the musculoskeletal system and connective tissue
MACI®	Matrix-applied characterised autologous cultured chondrocytes	Other drugs for disorders of the musculoskeletal system/M09AX02	Fractures, Cartilage	Diseases of the musculoskeletal system and connective tissue
Spherox®	Spheroids of human autologous matrix-associated chondrocytes	Other drugs for disorders of the musculoskeletal system/M09AX02	Cartilage Diseases	Diseases of the musculoskeletal system and connective tissue
Holoclar®	*Ex vivo* expanded autologous human corneal epithelial cells containing stem cells	Ophthalmologicals/S01XA19	Stem Cell Corneal Diseases	Diseases of the eye and adnexa

**TABLE 2:** Design features of pivotal clinical trials for the approved advanced therapy medicinal products in the European Union.

ATMP clinical development			Gene therapy medicinal products	Somatic cell therapy medicinal products	Tissue engineered therapies	All types of therapies
Number of products		N	10	3	4	17
Number of indications per product		Mean (SD)	1.10 (0.32)	1 (0)	1 (0)	1.06 (0.24)
Total number of pivotal trials and studies		N	14	4	5	23
—		Mean (SD)	1.27 (0.65)	1.33 (0.58)	1.25 (0.5)	1.28 (0.57)
—		(min, Max)	(1, 3)	(1, 2)	(1, 2)	(1, 3)
Clinical trials		—	—	—	—	—
Phase 1		N (%)	0 (0)	0 (0)	0 (0)	0 (0)
Phase 1/2		N (%)	4 (28.57)	1 (25)	0 (0)	5 (21.74)
Phase 2		N (%)	3 (21.43)	0 (0)	1 (20)	4 (17.39)
Phase 2/3		N (%)	3 (21.43)	0 (0)	0 (0)	3 (13.04)
Phase 3		N (%)	4 (28.57)	3 (75)	3 (60)	10 (43.48)
Observational retrospective studies	N (%)		0 (0)	0 (0)	1 (20)	1 (4.35)
Randomization		—	—	—	—	—
No		N (%)	12 (85.71)	1 (25)	1 (20)	14 (60.87)
Yes 1:1		N (%)	0 (0)	1 (25)	4 (80)	5 (21.74)
Yes ≥2:1		N (%)	2 (14.29)	2 (50)	0 (0)	4 (17.39)
Control		—	—	—	—	—
Not controlled		N (%)	6 (42.87)	0 (0)	2 (40)	8 (34.78)
Placebo controlled		N (%)	0 (0)	2 (50)	0 (0)	2 (8.70)
Active controlled		N (%)	1 (7.14)	1 (25)	3 (60)	5 (21.74)
Historical control		N (%)	6 (42.87)	1 (25)	0 (0)	7 (30.43)
Other		N (%)	1 (7.14)	0 (0)	0 (0)	1 (4.35)
Blinding		—	—	—	—	—
Open label		N (%)	14 (100)	2 (50)	5 (100)	21 (91.30)
Single blind		N (%)	0 (0)	0 (0)	0 (0)	0 (0)
Double blind		N (%)	0 (0)	2 (50)	0 (0)	2 (8.70)
Blinding evaluation		—	—	—	—	—
Yes		N (%)	2 (14.28)	2 (50)	5 (100)	19 (82.61)
No		N (%)	12 (85.71)	2 (50)	0 (0)	4 (17.39)
Multicentric		—	—	—	—	—
No		N (%)	4 (28.57)	0 (0)	0 (0)	4 (17.39)
Yes		N (%)	10 (71.43)	4 (100)	5 (100)	19 (82.60)
Number of arms		—	—	—	—	—
1 arm		N (%)	11 (78.57)	1 (25)	1 (20)	13 (56.52)
2 arms		N (%)	2 (14.29)	3 (75)	3 (60)	8 (34.78)
3 arms		N (%)	1 (7.14)	0 (0)	1 (20)	2 (8.70)
Design		—	—	—	—	—
Parallel groups		N (%)	2 (14.29)	3 (75)	3 (60)	8 (34.78)
Single arm		N (%)	11 (78.57)	1 (25)	1 (20)	13 (56.52)
Other		N (%)	1 (7.14)	0 (0)	1 (20)	2 (8.70)
Main Outcomes		—	—	—	—	—
Final variable		N (%)	2 (14.28)	2 (50)	1 (20)	5 (21.74)
Intermediate variable		N (%)	12 (85.71)	2 (50)	4 (80)	18 (78.26)
Co-primary		N (%)	2 (14.28)	1 (25)	1 (20)	4 (17.39)
Composite variable		N (%)	1 (7.14)	0 (0)	0 (0)	1 (4.35)
Single variable		N (%)	11 (78.57)	3 (75)	4 (80)	18 (78.26)
Type of variable for main outcome		—	—	—	—	—
Qualitative		N (%)	13 (92.85)	3 (75)	1 (20)	17 (73.91)
Quantitative (discrete and continuous)		N (%)	2 (14.28)	1 (25)	4 (80)	7 (30.43)
Health related quality of life		—	—	—	—	—
No		N (%)	7 (50)	2 (50)	1 (20)	10 (43.48)
General questionnaires		N (%)	5 (35.71)	1 (25)	1 (20)	7 (30.43)
Specific questionnaires		N (%)	4 (28.57)	1 (25)	4 (80)	9 (39.13)
Prespecified previous analysis		—	—	—	—	—
Interim analysis		N (%)	11 (78.57	3 (75)	3 (75)	17 (73.91)
Final analysis type (primary analysis)		—	—	—	—	—
ITT		N (%)	10 (71.43)	3 (75)	5 (100)	18 (78.26)
mITT		N (%)	2 (14.28)	0 (0)	0 (0)	2 (8.69)
PP		N (%)	2 (14.28)	0 (0)	0 (0)	2 (8.69)
Hypothesis		—	—	—	—	—
Superiority		N (%)	1 (7.14)	3 (75)	1 (20)	5 (21.74)
Non-inferiority		N (%)	0 (0)	0 (0)	2 (40)	2 (8.7)
Other		N (%)	13 (92.85)	1 (25)	2 (40)	16 (69.56)
Mean time for the main phase (months)		Mean (SD)	11.5 (9.30)	70.50 (91.22)	24 (9.80)	35.33 (31.08)
Ongoing at the time of the MAA submission (final data for primary outcome measure)		—	—	—	—	—
Yes		N (%)	8 (57.14)	3 (75)	1 (25)	12 (57.14)
No		N (%)	6 (42.86)	1 (25)	3 (75)	10 (47.62)
Population		—	—	—	—	—
Population randomized/enrolled		N	1,065	798	543	2,406
—		Median (Q25 - Q75)	22 (18.75–106.5)	134.5 (27–437)	104 (88.50–131)	75 (22–118)
—		(min, Max)	(5, 437)	(17, 512)	(75, 144)	(5, 512)
Population on intervention arm		N	797	495	254	1,546
—		Median (Q25 - Q75)	21.5 (11.5–93.75)	68.5 (20.25–282.5)	64.5 (53.25–72.75)	41 (16.25–93.75)
—		(min, Max)	(5, 296)	(17, 341)	(52, 73)	(5, 341)
Population on control arm		N	151	416	183	750
—		Median (Q25, Q75)	75.5 (NA)	140 (105–171)	61 (50–72)	88.50 (27.5–140.5)
—		(min, Max)	(10, 141)	(105, 171)	(50–72)	(10, 171)
Population on safety set		N	933	780	439	2,152
—		Median (Q25 -Q75)	22.5 (13.5, 93.75)	128.5 (25.75–430.75)	110 (81.75–137.5)	63.5 (20–118)
—		(min, Max)	(5, 419)	(17, 506)	(75, 144)	(5, 419)
Age of adult population (years)		Mean (SD)	54.29 (9.24)	52.77 (16.67)	37.14 (5.56)	47.84 (18.45)
Age of paediatric population (years)		Mean (SD)	6.15 (8.26)	NA	NA	6.15 (8.26)
Sex		—	—	—	—	—
Female		N (%)	443 (47)	191 (30.31)	231 (42.54)	865 (37.53)
Male		N (%)	498 (53)	630 (76.73)	312 (57.45)	1,440 (62.47)
Location of the pivotal clinical trial		—	—	—	—	—
United States		N (%)	9 (64.28)	1 (25)	0 (0)	10 (43.48)
Europe		N (%)	10 (71.42)	3 (75)	5 (100)	18 (78.26)
Canada		N (%)	5 (35.71)	1 (25)	0 (0)	6 (26.09)
Others		N (%)	7 (50)	3 (75)	0 (0)	10 (43.48)
Previous treatments		—	—	—	—	—
Yes and No		N (%)	1 (7.14)	0 (0)	0 (0)	1 (4.35)
No		N (%)	3 (21.74)	0 (0)	2 (40)	5 (21.74)
Yes		N (%)	10 (65.21)	2 (50)	3 (60)	15 (65.22)

ITT: intended to treat; mITT: modified intended to treat; NA: not applicable; PP: per protocol set; Zynteglo pooled analysis (Studies HGB-204, HGB-205 and LFT-303) was counted as one pivotal study; Holoclar retrospective study was counted as a pivotal study, since it was considered the main study which lead to the Marketing Authorisation of the product; The final analysis type (primary analysis) for TK0008 study of Zalmoxis was not available; The mean time for the main phase excludes Provenge (defined as ¨until disease progression or death¨) and TK0008 study for Zalmoxis; Age of adult population: data not available for TK0008 study for Zalmoxis; Age of paediatric population: data only available for Tecartus, Libmeldy, Kymriah and Strimvelis; Previous treatments: not applicable for Zalmoxis. For the Health related quality of life outcomes, the percentages can exceed 100% given that there might be multiple questioners for the same product (i.e., generic and disease-specific).

**TABLE 3:** Design features of pivotal clinical trials for the approved gene therapy medicinal products in the European Union.

Gene therapies	Glybera®			Imlygic®	Strimvelis®	Yescarta®	Kymriah®		Luxturna®	Zynteglo®		Zolgensma®	Tecartus®	Libmeldy®
CHMP Positive Opinion date	Jun-23-11			Oct-22-15	Apr-01-16	Jun-28-18	Jun-29-18		Set-20-18	Apr-26-19		Mar-26-20	Oct-15-20	Oct-15-20
Authorisation status/type	Withdrawn			Authorised	Authorised	Authorised	Authorised		Authorised	Authorised		Authorised	Authorised	Authorised
Type of authorisation	Under exceptional circumstances			Standard	Standard	Standard	Standard		Standard	Conditional		Conditional	Conditional	Standard
Clinical trial Acronym	CT-AMT-011–01	CT-AMT-011–02	CT-AMT-010–01	Study 005/05	Study AD1115611/Gene-ADA	ZUMA-1	Study B2202	Study C2201	AAV2-hRPE65v2-301/302	Studies HGB-204, HGB-205 and LFT-303	Studies HGB-207, HGB-212	Study CL-303 (STR1VE)	ZUMA-2	Study 201,222
Phase	II/III	II/III	II/III	III	I/II	I/II	II	II	III	I/II	III	III	II	I/II
Randomization	No	No	No	2:1	No	No	No	No	2:1	No	No	No	No	No
Control	Non-controlled	Non-controlled	Non-controlled	Active control	Historical control	Historical control	Historical control	Historical control	Delayed-intervention control group	Non-controlled	Non-controlled	Historical control	Non-controlled	Historical control
Blinding design	Open label	Open label	Open label	Open label	Open label	Open label	Open label	Open label	Open label	Open label	Open label	Open label	Open label	Open label
Blinding evaluation	No	No	No	No	No	No	No	No	Yes	No	No	No	Yes	No
Multicentric	Single-centre	Dual-centre	Single-centre	Multicentric	Single-centre	Multicentric	Multicentric	Multicentric	Dual-centre	Multicentre	Multicentre	Multicentre	Multicentre	Single-centre
Number of arms	Three	One	One	Two	One	One	One	One	Two	One	One	Two	One	One
Design	Parallel arms (dose range)	Single arm	Single arm	Parallel arms	Single arm	Single arm	Single arm	Single arm	Parallel arms	Single arm	Single arm	Single arm	Single arm	Single arm
Main Outcomes	Intermediate and single variable	Intermediate and single variable	Intermediate and composite variable	Intermediate and single variable	Final and single variable	Intermediate and single variable	Intermediate and single variable	Intermediate and single variable	Intermediate and single variable	Intermediate and single variable	Intermediate and single variable	Final and co-primary variable	Intermediate and single variable	Intermediate and co-primary variable
Type of variable for main outcome	Qualitative	Qualitative	Qualitative	Qualitative	Qualitative	Qualitative	Qualitative	Qualitative	Quantitative	Qualitative	Qualitative	Qualitative	Qualitative	Qualitative and Quantitative
Health related quality of life	No	General questionnaire	No	Specific questionnaire	No^a^	No	General questionnaire	General and specific questionnaires	No	No	General and specific questionnaires	No	General questionnaire	Specific questionnaire
Prespecified previous analysis	Interim analysis	Interim analysis	N/A	Interim analysis	None	Interim analysis	Interim analysis	Interim analysis	None	Interim analysis	Interim analysis	Interim analysis	Interim analysis	Interim analysis
Final analysis type (primary efficacy analysis)	ITT	ITT	ITT	ITT	ITT	mITT	PP	PP	ITT	ITT	ITT	ITT	mITT	ITT
Hypothesis	Description of efficacy of intervention	Description of efficacy of intervention	Description of efficacy of intervention	Superiority over an active control	Superiority over historical control group	Intervention compared to historical control	Description of efficacy of intervention	Intervention compared to historical control	Intervention compared non-intervention (natural history)	Description of efficacy of intervention	Description of efficacy of intervention	Superiority versus natural observation study	Description of efficacy of intervention	Superiority versus natural history cohort (or untreated sibling when available)
Mean time for the main phase (months)	3	3	3	12	36	12	3	12	12	12	12	14	3	24
Ongoing at the time of the MAA submission (final data for primary outcome measure)	Yes	Yes	No	No	No	Yes	Yes	Yes	No	Two studies ongoing	Yes	Yes	No	No
Population	—	—	—	—	—	—	—	—	—	—	—	—	—	—
Population randomised/enrolled	22	5	18	437	12	111	92	147	31	22	19	22	105	22
Population on intervention arm	14	5	8	296	12	101	75	99	21	22	10	22	92	20
Population on control arm	NA	NA	NA	141	NA	NA	NA	NA	10	NA	NA	NA	NA	NA
Population on safety set	14	5	8	419	12	101	75	99	29	23	14	22	92	20
Age of population (years)	—	—	—	—	—	—	—	—	—	—	—	—	—	—
Mean	45.6	41.8	N/A	63.07	1.7	56.3	12	54	N/A	N/A	N/A	0.31	65	3.6
Sex														
Female	9	1	N/A	250	5	33	32	36	18	15	6	12	15	11
Male	5	4	N/A	187	7	68	43	63	13	7	5	10	77	9
Geographic region	—	—	—	—	—	—	—	—	—	—	—	—	—	—
North America	X	X	—	X	—	X	X	X	X	X	X	X	X	—
Europe	—	—	X	X	X	X	X	X	—	X	X	—	X	X
Others	—	—	—	X	X	X	X	X	—	X	X	—	—	—
Previous treatments	Yes	Yes	Yes	Yes/No	Yes	Yes	Yes	Yes	No	Yes	Yes	No	Yes	No

ITT: intended to treat; mITT: modified intended to treat; NA: not applicable; N/A: not available; PP: per protocol set.

a Not at the time of the submission. The HRQoL objective applied to the long-term follow-up (4–8 years after gene therapy) only.

**TABLE 4:** Design features of pivotal clinical trials for the approved cell and tissue engineered therapy medicinal products in the European Union.

	Cell therapies				Tissue therapies				
	Provenge®	Zalmoxis®		Alofisel®	ChondroCelect®	Holoclar®	MACI®	Spherox®	
CHMP Positive Opinion date	Jun-12-13	Jun-23-16		Dec-14-17	Jun-25-09	Mar-06-13	Apr-25-13	May-18-17	
Authorisation status	Withdrawn	Withdrawn		Authorised	Withdrawn	Authorised	Withdrawn	Authorised	
Type of authorisation	Standard	Conditional marketing authorisation		Standard	Standard	Conditional	Standard	Standard	
Clinical trial Acronym	9902B (IMPACT)	TK007	TK008	ADMIRE-CD	TIG/ACT/01&EXT′	HLSTM01	SUMMIT	Cod 16 HS 14	Cod 16 HS 13
Phase	III	I/II	III	III	III	Observational retrospective study	III	II	III
Randomization	2:1	No	3:1	1:1	1:1	No	1:1	1:1:1	1:1
Control	Placebo	Historical control^a^	Active treatment	Placebo	Active treatment	Non-controlled	Active treatment	Non-controlled	Active treatment
Blinding	Double-blind	Open-label	Open-label	Double-blind	Open-label	Open-label	Open-label	Open-label	Open-label
Blinding evaluation	Yes	No	No	Yes	Yes	Yes	Yes	Yes	Yes
Multicentric	Multicentric	Multicentric	Multicentric	Multicentric	Multicentric	Dual-centre	Multicentric	Multicentric	Multicentric
Number of arms	Two	One	Two	Two	Two	One	Two	Three	Two
Design	Parallel groups	Single arm	Parallel groups	Parallel groups	Parallel groups	Retrospective case-series	Parallel groups	Single arm (three doses)	Parallel groups
Main Outcomes	Final and single variable	Intermediate and single variable	Intermediate and single variable	Final and co-primary variable	Intermediate and co-primary variable	Final and single variable	Intermediate and single variable	Intermediate and single variable	Intermediate and single variable
Type of variable for main outcome	Qualitative	Qualitative	Quantitative	Qualitative	Quantitative	Qualitative	Quantitative	Quantitative	Quantitative
Health related quality of life	No	No	General questionnaire	Specific questionnaire	Specific questionnaire	No	General and Specific questionnaire	Specific questionnaire	Specific questionnaire
Prespecified previous analysis	Interim analysis	None	Interim analysis	Interim analysis	None	NA	Interim analysis	Interim analysis	Interim analysis
Final analysis type (primary efficacy analysis)	ITT	ITT	NA	ITT	ITT	ITT	ITT	ITT	ITT
Hypothesis	Superiority over placebo	Description of efficacy of intervention	NA	Superiority over placebo	Non-inferiority vs SOC	Exploratory	Superiority over SOC	Superiority over baseline for the three dose groups	Comparison with baseline and non-inferiority/superiority with SOC
Duration of the main phase (months)	Until disease progression or death	135	NA	6	36	NA	24	12	24
Ongoing at the time of the MAA submission (primary completion)	No	No	Yes	No	No	NA	Yes	Yes	Yes
Population	—	—	—	—	—	—	—	—	—
Population enrolled	512	57	17	212	118	NA	144	75	102
Population on intervention arm	341	30	17	107	57	104	72	73	52
Population on control arm	171	140	Not known	105	61	NA	72	NA	50
Population on Safety set	506	52	17	205	118	NA	144	75	102
Age of population	—	—	—	—	—	—	—	—	—
Mean	71	49	N/A	38.3	33.9	46.8	34	34	37
Sex	—	—	—	—	—	—	—	—	—
Female	NA	30	N/A	161	42	24	51	53	61
Male	512	22	N/A	96	76	80	93	22	41
Geographic region	—	—	—	—	—	—	—	—	—
North America	X	—	—	—	—	—	—	—	—
Europe	—	X	X	X	X	X	X	X	X
Others	—	X	X	X	—	—	—	—	—
Previous treatments	Yes	NA	NA	Yes	Yes	Yes	No	No	No

ITT: intended to treat; NA: not applicable; N/A: not available; SOC: standard of care.

a Upon assessment of the TK007 data and as only limited data from the TK008 study were available, the applicant was asked to perform a comparison of the MM-TK treated patients (TK007 and TK008 combined) with results from suitable historical controls.

Regarding the design of the studies, 13 (56.52%) were Phase 2/3 and Phase 3 trials, 9 (39.13%) were Phase 1/2 or Phase 2 trials, and 1 (4.35%) was a retrospective study. For all types of therapies, 8 (34.78%) trials were non-controlled, 7 (30.44%) where active- or placebo-controlled, and 7 (30.43%) used an historical control as comparator. Differences were observed between gene and non-gene therapies (Supplementary Table S1 in Supplementary material). Six (42.87%) gene therapy studies were non-controlled and 6 (42.87%) used a historical control, whilst cell and tissue therapies studies were mainly controlled (66.66%). A total of 14 (60.87%) studies were not randomized. Similarly, differences in the existence of randomization between gene and non-gene therapies studies were also observed. Most of the studies for gene products lacked randomisation (85.71%), whereas this was present in 75% of the cell therapies studies and 80% of the tissue therapies studies. A total of 21 (91.30%) were open-label studies; all gene and tissue therapy studies were open-label, and this was also the approach for 50% of cell products trials. However, there is a difference in the blinding evaluation of the relevant endpoints between gene and non-gene therapy studies, as such evaluation is mostly absent in the case of gene therapies (85.71%) but is present in the case of cell and tissue engineered therapies (50% for cell therapy studies and 100% tissue engineered therapy studies). A total of 13 (56.52%) studies were single-arm trials and 10 (43.48%) had two or more arms. A difference in the number of arms between gene and non-gene therapy studies was also observed, where single-arm studies comprised 78.57% of total trials for gene therapy products versus the two- or three-arm designs present in 75% of cell therapy studies and 80% of tissue therapy studies. Accordingly, there are some differences in the design between gene and non-gene therapies studies, mainly in the parallel designs for cell and tissue engineered therapy studies versus single-arm designs for gene therapy studies. Of all studies analysed, 19 (82.60%) were multicentric.

Regarding the methodology used in these pivotal studies, 16 (69.56%) of the studies did not use a superiority or non-inferiority hypothesis but an alternative premise, e.g., comparison with historical controls. There is a difference between gene and non-gene therapies studies, where this type of alternative premises was mainly used for gene therapies trials (92.85%), while standard superiority or non-inferiority tests were used more frequently for cell and tissue engineered therapies trials (75 and 60%, respectively). To evaluate the primary objective, 18 (78.26%) of the trials used an intermediate and single main variable, which was mainly qualitative (73.91%). Final and quantitative variables were used in 5 (21.74%) and 7 (30.43%), respectively, which represents a smaller proportion (Table 5). Of these confirmatory studies, 18 (78.26%) used the intention-to-treat (ITT) principle in assessing the primary efficacy, 2 (14.28%) gene therapy trials used modified intention-to-treat (mITT) and 2 (14.28%) used per protocol set (PP). A total of 16 (69.56%) analysed studies included HRQoL questionnaires, 9 (39.13%) of those being disease-specific. No differences were observed in the type of HRQoL questionnaires between gene and non-gene products studies, i.e., generic versus disease-specific variables.

**TABLE 5:** Primary clinical variables of pivotal clinical trials for the approved ATMPs in the European Union.

Type of product	Product	Type of target disease	Intermediate (I) or final (F) variable	Primary variable description
GTMP	Kymriah (ALL)	Haematological malignancies	I	Overall remission rate, which included CR and CR with incomplete blood count recovery
	Kymriah (DLBCL)	Haematological malignancies	I	Overall response rate defined as the proportion of patients with a BOR of CR or PR, where the BOR was defined as the best disease response recorded from tisagenlecleucel until progression disease or start of new anticancer therapy
	Yescarta	Haematological malignancies	I	Objective response rate, defined as a CR or PR per the revised International Working Group Response Criteria for Malignant Lymphoma as determined by study investigators
	Tecartus	Haematological malignancies	I	Objective response rate, defined as CR or PR using central assessment per Lugano Classification
	Imlygic	Solid tumour	I	Durable response rate was defined as the percentage of participants with a CR or PR maintained continuously for at least 6 months from the time the objective response was first observed and initiating within 12 months of starting therapy as assessed by the Endpoint Assessment Committee
SCTMP	Provenge	Solid tumour	F	Overall survival defined as time from randomization to death due to any cause was analysed for the ITT population
GTMP	Glybera	Inherited monogenic diseases	I	Reduction in fasting plasma triglycerides (median of baseline vs median of week 3–12 post AMT-011) ≥ 40%
				Achievement of 40% reduction of median fasting triglycerides concentrations 12 weeks after treatment with AMT-011
				Reduction in individual median fasting plasma triglyceride levels of ≤10 mmol/L concurrent with a low-fat diet, or 40% reduction, concurrent with a low-fat diet
	Strimvelis	Inherited monogenic diseases	F	Survival at 3 years post-gene therapy
	Luxturna		I	Subject’s bilateral performance (no eye patching) on the mobility test, as measured by a change score, 1 year following vector administration as compared to a subject’s Baseline bilateral mobility test performance
	Zynteglo	Inherited monogenic diseases	I	The proportion of subjects who meet the definition of transfusion independence (TI). TI is defined as a weighted average Hb ≥ 9 g/dl without any packed red blood cells transfusions for a continuous period of ≥12 months at any time during the study after drug product infusion
	Zolgensma	Inherited monogenic diseases	F/Co-primary	Proportion of patients that achieve functional independent sitting for at least 30 s at the 18 months of age study visit. It is defined by the Bayley Scales of Infant and Toddler Development (Version 3), confirmed by video recording, as a patient who sits up straight with head erect for at least 30 s
				Survival at 14 months of age
	Libmeldy	Inherited monogenic diseases	I/Co-primary	Total Gross Motor Function Measure score 2 years after treatment was the primary endpoint
				The co-primary endpoint was the ARSA activity
TEP	Chondrocelect	Condrophaties	I/Co-primary	Histomorphometry on end point biopsies at 12 months post-surgery and overall Histology Assessment on First Subscale of ICRS II Score
				Change from Baseline in Overall Knee Injury and Osteoarthritis Outcome Score at 12–18 Months
	MACI	Condrophaties	I	Change from Baseline to Week 104 for the Participant’s Knee Injury and Osteoarthritis Outcome Score Pain and Function (Sports and Recreational Activities) Scores
	Spherox	Condrophaties	I	Change of overall Knee Injury and Osteoarthritis Outcome Score from baseline to final assessment determined for each dosage group and between the dosage groups
				Change of overall Knee Injury and Osteoarthritis Outcome Score from baseline to final assessment compared between intervention arm and comparator
SCTMP	Zalmoxis	Adjunctive treatment in haploidentical haematopoietic stem cell transplantation	I	Proportion of patients who achieved immune reconstitution, empirically defined a priori as an absolute CD3^+^ cell count of 100/μL or more for two consecutive observations (and/or CD4^+^ cells ≥50/μL and/or CD8^+^ cells ≥50/μL)
				Disease-free survival measured from the date of randomization until the date of relapse (or progression), or death from any cause, whichever occurs first
	Alofisel	Complex perianal fistula(s)—Crohn’s disease	F/Co-primary	Combined remission of perianal fistulising Crohn’s disease and absence of collections >2 cm of the treated fistula confirmed by MRI images, at week 24. Remission was defined as clinical closure of external openings that were draining at baseline despite gentle finger compression
TEP	Holoclar	Limbal stem cell deficiency	F	Composite endpoint of the rate of patients with a successful transplantation at 12 months post-intervention, based on the co-presence of clinical signs

**Intermediate variable**: a clinical endpoint such as measure of a function or of a symptom (disease-free survival, angina frequency, exercise tolerance) but is not the ultimate endpoint of the disease, such as survival or the rate of irreversible morbid events (stroke, myocardial infarction).

**Final variable:** describes a valid measure of clinical benefit due to treatment: the impact of treatment on how a patient feels, functions and survives. It is clinically relevant, sensitive (responsive to change) and is both accepted and used by physicians and patients. Clinical endpoints may be a clinical event (e.g. mortality) a composite of several events, a measure of clinical status, or health related quality of life (HRQoL) [*Ref:* EUnetHTA, 2015a, b)*. Guideline on Endpoints used for Relative Effectiveness Assessment of pharmaceuticals: Clinical endpoints.* *https://www.eunethta.eu/wp-content/uploads/2018/01/Clinical-endpoints.pdf* ].

ARSA, arysulfatase A enzyme; CR, complete response; GTMP, gene therapy medicinal product; ITT, intended to treat; PR, partial response; SCTMP, somatic cell therapy medicinal product; TEP, tissue engineered medicinal product.

The mean (SD) time for the main phase of the trial was 35.33 (31.08) months, approximately 1 year for the gene therapies and more than 2 years for cell and tissue engineered therapies. A total of 12 (57.14%) studies were ongoing at the time of submission, meaning that the final data collection for primary outcome measuring was not completed. Globally, 17 (73.91%) of the studies had a prespecified interim analysis, with similar proportion among the three types of ATMPs (75–78.57%).

Regarding the overall population size and location of these studies, the median (IQR 25–75) number of patients enrolled in the analysed ATMPs pivotal clinical trials was 75 (22–118). The mean ± SD age of the adult population included in these confirmatory trials was 48 ± 18.45 years old. There is no sufficient data to establish a mean ± SD age for paediatric populations. The sex distribution is higher for males (62.47%) than for women (37.53%). The analysed clinical trials were equally performed in both women and men, but the overall sex distribution was higher for males due to Provenge^®^’s indication, i.e., treatment of metastatic castration-resistant prostate cancer. The median (IQR 25–75) sample size in the intervention arm was 41 (16–94) patients and 63 (20–118) for the safety set. More than half of participants in these clinical trials had received previous treatments (65.22%). From the 23 pivotal studies analysed, 18 included sites located in the EU (78.26%), and 10 (43.48%) in the United States of America (US) or in other regions, such as Israel, Japan or Australia.

## Discussion

Clinical research on ATMPs has increased during the last few years (Alamo et al., 2019). The introduction of ATMPs and the long-term expectancy of their benefit adds a new challenge for the regulatory agencies. In the present study, we aimed to describe the most relevant methodological features of the clinical trials that have driven ATMPs to their approval. The major findings reveal that the pivotal studies of currently approved advanced therapies typically share the following characteristics: 1) they are small, open-label, non-randomised, single-arm studies without control or using historical ones, and 2) intermediate and single variables are used to evaluate the primary efficacy outcome. In addition, this type of designs is more common for gene therapies than for cell and tissue therapies.

Hanna *et al* previously reported the methodological characteristics of clinical trials assessing ATMPs in an early development phase based on clinical trials registries (Hanna et al., 2016). The results showed very similar characteristics to those found in this study such as small sample size, non-randomised trials, single-arm trials, surrogate endpoints, and adaptive designs. Coppens *et al.*, also reported that the level of scientific evidence required for the approval might differ among different regulatory agencies (Coppens et al., 2018). Elsallab *et al.* showed that clinical trials of ATMPs did not meet the same strict standards for clinical evidence that were applied to other biologicals submissions (Elsallab et al., 2020). This previously reported data, together with the results of the present study, highlight the limited clinical evidence upon which the authorisation of most ATMPs is based. Of these approved ATMPs, it was considered that eleven (64.70%) had sufficient data for a full MA, while for the remaining six products, five (29.41%) obtained a conditional approval and one (5.88%) was granted with a MA under exceptional circumstances.

The low disease prevalence, the disease severity and burden, the lack or scarce availability of disease-modifying treatments, the patient population’s heterogeneity and the strong presence of paediatric patient populations comprise some of the factors that could contribute to this type of designs.

The type of target diseases has been one of the key factors that might have given more flexibility in terms of level of evidence required for the MA. Our analysis shows that these designs are more commonly used for the development of gene therapy products, which target orphan diseases such as hematologic cancers or rare inherited monogenic disorders (40 and 60% of approved gene therapies, respectively), usually with unmet medical needs. Gene therapies were mainly authorised after conducting a single open-label study, usually non-randomised and non-controlled or using historical controls, and only few of them being Phase III studies. By contrast, tissue therapies trials consisted of Phase III studies controlled with the standard of care, and two out of three cell therapy trials conducted placebo-controlled studies. The approved tissue therapies primarily cover products for articular cartilage damage or prostate cancer, which are relatively common among the overall population and with several treatments available. Moreover, the target population might have also contributed to these alternative designs for gene therapies products, given that 60% of approved gene therapies target paediatric population, while all of the tissue and cell therapies target adults. The targeted paediatric diseases are life-threatening or with a huge impact on patients’ and caregivers’ quality-of-life, and randomised, controlled trials could have posed ethical concerns, as well as recruitment issues.

It is noteworthy to mention that different types of historical controls were used to compare the efficacy of the intervention: historical references from retrospective studies and retrospective databases, prospective natural history cohorts’ studies, untreated sibling data and within-subject comparison between pre- and post-treatment assessments (Hassan et al., 2012; European Medicines Agency, 2021; Maude et al., 2018; Crump et al., 2017). The current EMA guideline states that orphan products are assessed according to the same standards as those for other products but considering their limitations due to low patient recruitment (European Medicnes Agency, 2006). While the same guideline states that most orphan drugs and paediatric indications submitted for regulatory approval are based on randomised controlled trials and deviation from such standards is uncommon, in the case of the current approved ATMPs, alternative approaches as historical controls were frequently used, i.e., Strimvelis^®^, Kymriah^®^, Luxturna^®^, Zolgensma^®^ and Libmeldy^®^.

On the other hand, the line of treatment is another factor that might have justified these types of designs so far. As an example of the approved ATMPs, CAR-T therapies are indicated at least as a third-line therapy for relapsed or refractory cancer patients. The four pivotal studies conducted for these products were non-controlled, open-label, Phase II studies where the intervention arm was compared to a historical control. After the approval of the aforementioned therapies, the EMA has published recommendations on clinical considerations on CAR-T-cell product development (European Medicnes Agency, 2020), where it is stated that randomized controlled trial design should be followed even for those cases of late-stage refractory disease. It will be interesting to see how these recommendations are implemented in the near future.

Another important factor observed in the studied designs is the use of surrogate variables instead of a clinically relevant final endpoint. Intermediate endpoints can be used as a primary endpoint for MA, especially when there is a high unmet need, when clinical events are rare/delayed in slowly progressive diseases and a very long follow-up is needed for their assessment, and for rare and/or life-threatening diseases with no therapeutic alternative available (EUnetHTA, 2015b; ICH, 1998a). In the case of all approved gene therapies that target cancer diseases, the proportion of patients with objective overall response rate (ORR) was used as the intermediate primary variable, unlike cell therapy trials that used overall survival (OS) as a final endpoint. OS analysis usually requires a large sample size, a long follow-up and should be evaluated in a randomised, control trial to avoid cofounding factors due to the switch-over of control to intervention or subsequent therapies (Gutman et al., 2013; Pazdur, 2008). However, ORR has been the most commonly used surrogate endpoint in support of accelerated/conditional approvals, but also of standard approvals, since it is directly attributable to a drug’s effect, providing an accurate assessment in single-arm trials conducted in patients with refractory tumours (Food and Drug Administration, 2018a). On the other hand, for gene therapies targeting inherited monogenic diseases, biomarkers were commonly used to predict changes in the desired clinical endpoints, and at least one of the pivotal studies included HRQoL outcomes. Exceptionally for other products, a novel clinical meaningful endpoint, i.e. Luxturna® (Russell et al., 2017), or survival as a final primary outcome were used, i.e. Zolgensma® (Del Rosario et al., 2020; Cech et al., 2012).

These types of non-robust designs for new drugs in areas of high unmet medical need are mainly justified on the basis of ethical reasons, based on the potential life-saving opportunities or quality of life improvement for patients who may not survive or will progress rapidly until robust clinical data is available. On the other hand, the difficulties of conducting standard clinical developments with orphan drugs are well-recognised, and single small trials using alternative approaches have been the basis for numerous MAA in the recent years (Blin et al., 2020; Micallef and Blin, 2020; Picavet et al., 2013; Pontes et al., 2018). This regulatory flexibility sometimes comes at the cost of having a less comprehensive clinical data, and in consequence, greater uncertainty about the product’s benefit-risk balance at the time of MA (Iglesias-Lopez et al., 2021b). In addition, since the introduction of the adaptive pathway concept, the shift towards accelerated clinical developments has also been associated with an intrinsic uncertainty on effectiveness and safety, which can result in promising Phase II results but an unsuccessful Phase III or post-marketing studies (Pharma Intelligence, 2019; Novartis press release, 2021a, b). This highlights the possibility for a patient to receive an early-authorised treatment without meaningful clinical benefits and with exposure to its adverse effects, missing clinical opportunities, and wasting healthcare system resources (Ermisch et al., 2016).

The speeding up access to new drugs is achieved by putting aside traditional Phase III clinical trials in favour of post-marketing evidence generation. This fact is translated into the need to perform long and extensive post-marketing studies, where the costs of evidence generation as well as the costs of therapy are likely to be transferred from the MA holder to healthcare systems (Ermisch et al., 2016; Joppi et al., 2016). It is known, that this post-authorisation commitments can be challenging due to the long-term follow-up, which may lead to delays to complete the studies, and given that patients are more reluctant to participate in a post-marketing trial with all its constraints, if the medicine is already available, above all in those cases where the trial includes randomization (Joint briefing paper, 2015).

Costly treatments with high uncertainties in regard to its benefits, translates to a complex evaluation by the Health Technology Assessment bodies (HTAb), as well as there is industry pressure for corporate pharma and its investors to ensure sustainability in drug development.

Several detailed methodological recommendations for clinical trial designs have been launched to address the shortcomings of carrying out studies in small population (Day et al., 2018; ASTERIX project, 2021; IDEAL project, 2021; Friede et al., 2018) and examples of effective use of a historical control have also been reported (Mulberg et al., 2019). Multi-arm designs and platform designs sharing where a common control is shared have been raised as a potential solution (International Rare Diseases Research Consortium, 2016; Food and Drug Administration, 2018b). Comparator data can also be taken from pragmatic trials, observational studies or registries, but ensuring its quality (EUnetHTA, 2015a). In addition, real world data plays a key role to provide sufficient therapeutic evidence for these type of therapies and efforts are being made for a better use of registries (European Medicines Agency, 2017).

Methodological and clinical guidelines for a specific medical condition is an effective manner of obtaining regulatory guidance and providing a predictable decision-making regulatory framework. Given that ATMPs are innovative and more complex than traditional pharmaceuticals or other biological drugs, some specific requirements related to the study design and methodology, study population, safety, dose selection, as well as preclinical and product controls need to be considered for the development of these therapies. The FDA has launched several guidelines for the development of ATMPs aimed at certain types of conditions based on the acquired experience of the current approved advanced therapies. These guidelines address the point of uncontrolled designs and the need of more robust study designs in order to provide proper evidence of efficacy (Food and Drug Administration, 2020a; Food and Drug Administration, 2020b; Food and Drug Administration, 2021). Although still limited, with the current experience of the approved ATMPs in the EU, the EMA has started to launch new recommendations on the types of study designs and methodologies that can support the MA more robustly (European Medicnes Agency, 2020). This fact might lead to a switch on the current trend used in clinical designs based on uncontrolled pivotal studies or with historical control comparisons to randomised-controlled trials.

The limitations of this study are the small sample size and the fact that further analysis, once more therapies are approved, is required to determine with greater accuracy the most common clinical design and methodology for ATMPs, as well as to elucidate the potential differences between gene therapy trials versus cell and tissue therapy trials. Another limitation is that approved ATMPs have not been compared to other approved medicines. Nevertheless, this is an exhaustive study that evaluates the pivotal trials for approved ATMPs.

## Conclusion

The results of our study show that most authorised ATMPs are based on small, open-label, uncontrolled and single-arm pivotal trials using single and intermediate variables to evaluate outcomes. ATMPs are innovative therapies that mainly target orphan diseases and high unmet medical needs. This fact has led to certain methodological. weaknesses in their pivotal clinical trials, which in turn has resulted in limited data to robustly assess the benefit/risk of the product. A gradual shift towards the production of more methodologically sound randomized-controlled trials is expected to better define the benefit and the therapeutic added value of ATMPs.

## Data Availability

The raw data supporting the conclusion of this article will be made available by the authors, without undue reservation.

## References

[B3] Álamo J. E. Timón M. Gómez-Platero C. G. Abad C. D. González M. V. (2019). Clinical Trials of Advanced Therapy Investigational Medicinal Products in Spain: Preparing for the European Clinical Trials Regulation. Cell Gene Ther. Insights 5, 1431–1449. 10.18609/cgti.2019.147

[B4] ASTERIX project (2021). Advances in Small Trials dEsign for Regulatory Innovation and eXcellence. Available at: http://www.asterix-fp7.eu/ (Accessed August 28, 2021).

[B5] Blin O. Lefebvre M. N. Rascol O. Micallef J. (2020). Orphan Drug Clinical Development. Therapie 75, 141–147. 10.1016/j.therap.2020.02.004 32247678

[B7] Cech D. J. Martin S. T. (2012). Chapter 5-Evaluation of Function, Activity, and Participation," in Functional Movement Development Across the Life Span. Elsevier, 88–104. 10.1016/b978-1-4160-4978-4.00005-3

[B8] Coppens D. G. M. de Wilde S. Guchelaar H. J. De Bruin M. L. Leufkens H. G. M. Meij P. (2018). A Decade of Marketing Approval of Gene and Cell-Based Therapies in the United States, European Union and Japan: An Evaluation of Regulatory Decision-Making. Cytotherapy 20, 769–778. 10.1016/j.jcyt.2018.03.038 29730080

[B9] Crump M. Neelapu S. S. Farooq U. Van Den Neste E. Kuruvilla J. Westin J. (2017). Outcomes in Refractory Diffuse Large B-Cell Lymphoma: Results from the International SCHOLAR-1 Study. Blood 130, 1800–1808. 10.1182/blood-2017-03-769620 28774879 PMC5649550

[B10] Day S. Jonker A. H. Lau L. P. L. Hilgers R. D. Irony I. Larsson K. (2018). Recommendations for the Design of Small Population Clinical Trials. Orphanet J. Rare Dis. 13, 195. 10.1186/s13023-018-0931-2 30400970 PMC6219020

[B11] Del Rosario C. Slevin M. Molloy E. J. Quigley J. Nixon E. (2020). How to Use the Bayley Scales of Infant and Toddler Development. Arch. Dis. Child. Educ. Pract. Ed. 106, 108–112. 10.1136/archdischild-2020-319063 32859738

[B12] Elsallab M. Bravery C. A. Kurtz A. Abou-El-Enein M. (2020). Mitigating Deficiencies in Evidence during Regulatory Assessments of Advanced Therapies: A Comparative Study with Other Biologicals. Mol. Ther. Methods Clin. Dev. 18, 269–279. 10.1016/J.OMTM.2020.05.035 32637456 PMC7327881

[B13] Ermisch M. Bucsics A. Vella Bonanno P. Arickx F. Bybau A. Bochenek T. (2016). Payers' Views of the Changes Arising through the Possible Adoption of Adaptive Pathways. Front. Pharmacol. 7, 305. 10.3389/fphar.2016.00305 27733828 PMC5039228

[B14] EUnetHTA (2015a). Criteria for the Choice of the Most Appropriate Comparator(s) Summary of Current Policies and Best Practice Recommendations. Available at: https://eunethta.eu/wp-content/uploads/2018/03/Criteria_WP7-SG3-GL-choice_of_comparator_amend2015.pdf (Accessed April 19, 2021).

[B15] EUnetHTA (2015b). Guideline on Endpoints Used for Relative Effectiveness Assessment of Pharmaceuticals: Surrogate Endpoints. Available at: https://eunethta.eu/wp-content/uploads/2018/03/surrogate_endpoints.pdf .

[B16] European Medicines Agency (2021). Committee for Medicinal Products for Human Use (CHMP) Assessment Report: Libmeldy. Available at: https://www.ema.europa.eu/en/documents/assessment-report/libmeldy-epar-public-assessment-report_en.pdf .

[B18] European Medicines Agency (2017). Patient Registry Initiative- Strategy and Mandate of the Cross-Committee Task Force. Available at: www.ema.europa.eu/contact (Accessed July 23, 2021).

[B19] European Medicines Agency (2010). Reflection Paper on Classification of Advanced Therapy Medicinal Products. Available at: https://www.ema.europa.eu/en/documents/scientific-guideline/reflection-paper-classification-advanced-therapy-medicinal-products_en-0.pdf (Accessed March 6, 2020).

[B20] European Medicnes Agency (2006). Committe for Medicinal Products for Human Use (CHMP), Guideline on Clinial Trials in Small Populations Populations. Available at: https://www.ema.europa.eu/en/documents/scientific-guideline/guideline-clinical-trials-small-populations_en.pdf (Accessed March 28, 2021).

[B22] European Medicnes Agency (2020). Guideline on Quality, Non-clinical and Clinical Aspects of Medicinal Products Containing Genetically Modified Cells. Available at: https://www.ema.europa.eu/en/documents/scientific-guideline/guideline-quality-non-clinical-clinical-aspects-medicinal-products-containing-genetically-modified_en-0.pdf (Accessed February 2, 2021).

[B23] European Medicnes Agency (2007). Reflection Paper on Methodological Issues in Confirmatory Clinical Trials Planned with an Adaptive Design. London: EMEA Doc. Ref. CHMP/EWP/2459/02 http://www.emea.europa.eu (Accessed May 10, 2021).Available at:

[B24] Food and Drug Administration (2018a). Clinical Trial Endpoints for the Approval of Cancer Drugs and Biologics Guidance for Industry. Available at: https://www.fda.gov/Drugs/GuidanceComplianceRegulatoryInformation/Guidances/default.htmand/orhttps://www.fda.gov/BiologicsBloodVaccines/GuidanceComplianceRegulatoryInformation/Guidances/default.htm (Accessed April 11, 2021).

[B25] Food and Drug Administration (2020a). Human Gene Therapy for Hemophilia; Guidance for Industry. Available at: https://www.fda.gov/vaccines-blood-biologics/guidance-compliance- (Accessed February 2, 2021).

[B26] Food and Drug Administration (2021). Human Gene Therapy for Neurodegenerative Diseases; Draft Guidance for Industry. Available at: https://www.fda.gov/vaccines-blood-biologics/guidance-compliance- (Accessed February 2, 2021).

[B27] Food and Drug Administration (2020b). Human Gene Therapy for Retinal Disorders; Guidance for Industry. Available at: https://www.fda.gov/vaccines-blood-biologics/guidance-compliance- (Accessed February 2, 2021).

[B28] Food and Drug Administration (2018b). Master Protocols: Efficient Clinical Trial Design Strategies to Expedite Development of Oncology Drugs and Biologics Guidance for Industry DRAFT GUIDANCE. Available at: https://www.fda.gov/Drugs/GuidanceComplianceRegulatoryInformation/Guidances/default.htm (Accessed July 20, 2021).

[B30] Friede T. Posch M. Zohar S. Alberti C. Benda N. Comets E. (2018). Recent Advances in Methodology for Clinical Trials in Small Populations: the InSPiRe Project. Orphanet J. Rare Dis. 13, 186. 10.1186/s13023-018-0919-y 30359266 PMC6203217

[B31] Gutman S. I. Piper M. Grant M. D. (2013). Progression-Free Survival: What Does It Mean for Psychological Well-Being or Quality of Life? Rockville, MD: Agency for Healthcare Research and Quality (US). Available at: https://www.ncbi.nlm.nih.gov/books/NBK137763/ (Accessed April 11, 2021). 23678517

[B32] Hanna E. Rémuzat C. Auquier P. Toumi M. (2016). Advanced Therapy Medicinal Products: Current and Future Perspectives. J. Mark Access Health Pol. 4, 31036. 10.3402/jmahp.v4.31036 PMC4846788 27123193

[B33] Hassan A. Booth C. Brightwell A. Allwood Z. Veys P. Rao K. (2012). Outcome of Hematopoietic Stem Cell Transplantation for Adenosine Deaminase-Deficient Severe Combined Immunodeficiency. Blood 120, 3615–3626. 10.1182/blood-2011-12-396879 22791287

[B34] ICH (1998a). ICH Topic E8 General Considerations for Clinical Trials Step 5 Note for Guidance on General Considerations for Clinical Trials. Available at: http://www.emea.eu.int (Accessed April 11, 2021).

[B35] ICH (1998b). ICH Topic E9 Statistical Principles for Clinical Trials Step 5 Note for Guidance on Statistical Principles for Clinical Trials. Available at: http://www.emea.eu.int (Accessed March 27, 2021).

[B36] IDEAL project (2021). Integrated DEsign and AnaLysis of Clinical Trials in Small Population Groups. Available at: https://www.ideal.rwth-aachen.de/ (Accessed August 28, 2021).

[B37] Iglesias-López C. Agustí A. Obach M. Vallano A. (2019). Regulatory Framework for Advanced Therapy Medicinal Products in Europe and United States. Erratum in: Front Pharmacol 11, 766. 10.3389/fphar.2019.00921 PMC7261870 32523535

[B38] Iglesias-Lopez C. Obach M. Vallano A. Agustí A. (2021a). Comparison of Regulatory Pathways for the Approval of Advanced Therapies in the European Union and the United States. Cytotherapy 23, 261–274. 10.1016/j.jcyt.2020.11.008 33483292

[B55] Iglesias-Lopez C. Agustí A. Vallano A. Obach M. (2021b). Current landscape of clinical development and approval of advanced therapies. *Mol Ther Methods Clin Dev* 23, 606–618. 10.1016/j.omtm.2021.11.003 PMC8626628 34901306

[B39] International Rare Diseases Research Consortium (2016). Small Population Clinical Trials Task Force Workshop Report and Recommendations. Available at: https://www.irdirc.org/wp-content/uploads/2017/12/SPCT_Report.pdf (Accessed May 16, 2021).

[B42] Joint briefing paper (2015). "Adaptive Licensing” or “Adaptive Pathways”: Deregulation under the Guise of Earlier Access Executive. Available at: http://english.prescrire.org/Docu/DOCSEUROPE/ (Accessed April 19, 2021).

[B43] Joppi R. Gerardi C. Bertele' V. Garattini S. (2016). Letting post-marketing Bridge the Evidence gap: The Case of Orphan Drugs. BMJ 353, i2978. 10.1136/bmj.i2978 27335134

[B44] Maude S. L. Laetsch T. W. Buechner J. Rives S. Boyer M. Bittencourt H. (2018). Tisagenlecleucel in Children and Young Adults with B-Cell Lymphoblastic Leukemia. N. Engl. J. Med. 378, 439–448. 10.1056/nejmoa1709866 29385370 PMC5996391

[B45] Micallef J. Blin O. (2020). Orphan Drug Designation in Europe: A Booster for the Research and Development of Drugs in Rare Diseases. Therapie 75, 133–139. 10.1016/j.therap.2020.02.003 32156423

[B47] Mulberg A. E. Bucci-Rechtweg C. Giuliano J. Jacoby D. Johnson F. K. Liu Q. (2019). Regulatory Strategies for Rare Diseases under Current Global Regulatory Statutes: A Discussion with Stakeholders. Orphanet J. Rare Dis. 14 (1), 36. 10.1186/s13023-019-1017-5 30736861 PMC6368795

[B48] Novartis press release (2021a). Novartis’ Kymriah Fails to Meet Primary Goal in Non-hodgkin Lymphoma Trial. Available at: https://www.clinicaltrialsarena.com/news/novartis-kymriah-lymphoma-trial/ (Accessed August 27, 2021).

[B49] Novartis press release (2021b). Novartis Kymriah® Demonstrates Consistent Efficacy and Safety Outcomes in US Patients when Used in Real-World Setting | Novartis. Available at: https://www.novartis.com/news/media-releases/novartis-kymriah-demonstrates-consistent-efficacy-and-safety-outcomes-us-patients-when-used-real-world-setting (Accessed April 18, 2021).

[B50] Pazdur R. (2008). Endpoints for Assessing Drug Activity in Clinical Trials. Oncologist 13 (Suppl. 2), 19–21. 10.1634/theoncologist.13-S2-19 18434634

[B51] Pharma Intelligence (2019). Disappointing End for MolMed’s Zalmoxis Cell Therapy in EU. Available at: https://pink.pharmaintelligence.informa.com/PS140998/Disappointing-End-For-MolMeds-Zalmoxis-Cell-Therapy-In-EU (Accessed March 8, 2020).

[B52] Picavet E. Cassiman D. Hollak C. E. Maertens J. A. Simoens S. (2013). Clinical Evidence for Orphan Medicinal Products-A Cause for Concern? Orphanet J. Rare Dis. 8, 164. 10.1186/1750-1172-8-164 24131572 PMC3852769

[B53] Pontes C. Fontanet J. M. Vives R. Sancho A. Gómez-Valent M. Ríos J. (2018). Evidence Supporting Regulatory-Decision Making on Orphan Medicinal Products Authorisation in Europe: Methodological Uncertainties. Orphanet J. Rare Dis. 13, 206. 10.1186/s13023-018-0926-z 30442155 PMC6238348

[B54] Russell S. Bennett J. Wellman J. A. Chung D. C. Yu Z. F. Tillman A. (2017). Efficacy and Safety of Voretigene Neparvovec (AAV2-hRPE65v2) in Patients with RPE65-Mediated Inherited Retinal Dystrophy: a Randomised, Controlled, Open-Label, Phase 3 Trial. Lancet 390, 849–860. 10.1016/S0140-6736(17)31868-8 28712537 PMC5726391

