# Supplementary material for: Methodological Characteristics of Clinical Trials Supporting the Marketing Authorisation of Advanced Therapies in the European Union
Source: Front Pharmacol. 2021 Nov 29;12:773712. doi: 10.3389/fphar.2021.773712 (PMC8668425; doi:10.3389/fphar.2021.773712)
Supplement: Supplementary file 1 [file Table1.pdf]

Supplementary Material

1 Supplementary Tables

Table S1. Differences by type of advanced therapy (gene therapies versus cell and tissue therapies)

Design and methodology of the pivotal trial	Gene Therapies	Non-Gene Therapies
**Mean (SD) number of pivotal clinical trials**	1,27 (0,65)	1,14 (0,38)
**Phase of the trials**		
Phase 1, Phase 1/2, Phase 2 and retrospective trials	7	3
Phase 2/3 and Phase 3[1]	7	6
**Type of control**		
Non-controlled[2]	13	3
Placebo or active controlled	1	6
**Randomisation**		
Yes	2	6
No	12	3
**Blinding design**		
Open	14	7
Single or double	0	2
**Blinding evaluation**		
Yes	2	7
No	12	2
**Multicentre**		
Yes	11	9
No	3	0
**Number of arms**		
1 arm	11	2
≥ 2 arms	3	7
**Design**		
Parallel	2	6
Other[3]	12	3
**Type of objective**		
Superiority and non-inferiority	1	6
Other	13	3
**Main outcome**		
Final variable	2	3
Intermediate variable	12	6
**Type of primary variable**		
Number of quantitative variables	13	4
Number of qualitative variables	2	5
**Health-related quality of life variables**		
Yes	9	7
No	7	3

Sample size (n=17): Gene therapies (n= 10), non-gene therapies (n=7). Non-gene therapies comprise both cell and tissue engineered medicinal products. [1] Including 1 retrospective study; [2] Including historical controls and ¨other¨ studies; [3] Including single, crossover and ¨other¨ studies. SD: standard deviation.
